# Label-free electronic detection of peptide post-translational modification with functional enzyme-driven assay at the physical limit

**DOI:** 10.1016/j.xcrp.2024.101874

**Published:** 2024-08-21

**Authors:** Eleonora Macchia, Kim Björkström, Amit Tewari, Ville Eskonen, Axel Luukkonen, Amir Mohammad Ghafari, Lucia Sarcina, Mariapia Caputo, Natalia Tong-Ochoa, Kari Kopra, Fredrik Pettersson, Zahra Gounani, Luisa Torsi, Harri Härmä, Ronald Österbacka

**Affiliations:** 1 Physics and Center for Functional Materials, Faculty of Science and Engineering, Åbo Akademi University, 20500 Turku, Finland; 2 Department of Pharmaceutical Sciences, Università degli Studi di Bari Aldo Moro, 70125 Bari, Italy; 3 Chemistry of Drug Development, Department of Chemistry, University of Turku, 20500 Turku, Finland; 4 Dipartimento di Chimica, Università degli Studi di Bari Aldo Moro, 70125 Bari, Italy

**Keywords:** peptide detection, single-molecule detection, organic bioelectronics, single-molecule assay with large transistors, SiMoT, multivariate data processing

## Abstract

High-performance, ultra-sensitive, and universal protein post-translational modification (PTM) and protein-protein interaction (PPI) technologies are eagerly pursued in the pharmaceutical industry and bioanalytical research. Novel PTM and PPI detection methods outperform traditional assays in scope and scalability, enabling the collection of information on multiple biochemical targets. Detecting peptides and proteins at the single-molecule level is done by utilizing nanosized transducing elements and assaying solutions at very high analyte concentrations, in the nanomolar range or higher. Here, a proof of principle of a biosensing platform for single-molecule PTM detection is demonstrated. This platform is based on the single molecule with a large transistor (SiMoT) technology, encompassing a millimeter-sized electrolyte-gated organic field-effect transistor, for label-free PTM detection with a zeptomolar limit of detection. Sensitivity is improved 10^6^- to 10^12^-fold compared with mass-spectrometry and luminescence-based assay methods. A functional assay for detecting enzyme-driven peptide PTMs in the zeptomolar concentration range is demonstrated using multivariate data processing, opening the way for future applications to monitor PTMs.

## Highlights

• Electrolyte-gated organic field-effect transistor as a biosensing platform
• Detection of peptide single-site and enzyme-driven post-translational modifications
• Label-free, selective bioassay detection at the physical limit of single peptides

## Graphical abstract

**Figure:**
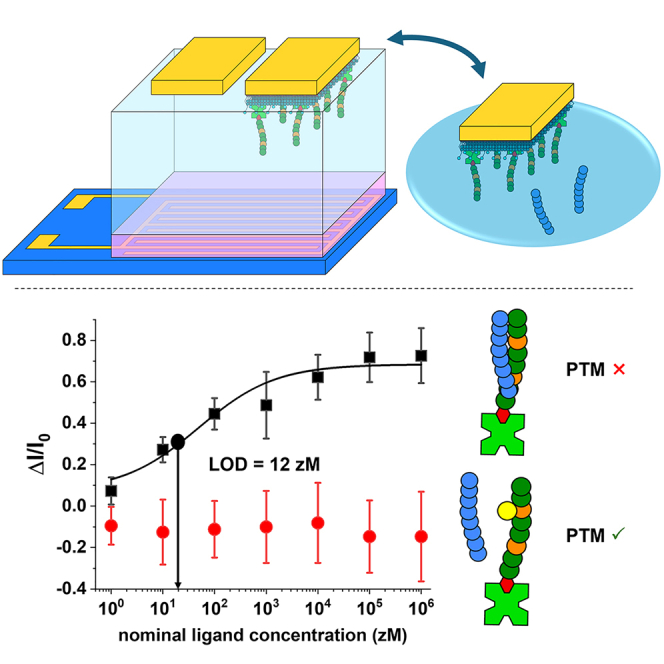

## Teaser

High-performance, ultra-sensitive, and universal protein post-translational modification (PTM) and protein-protein interaction (PPI) detection technologies are eagerly pursued in industry and research. Macchia, Björkström, and Tewari et al. demonstrate label-free, electronic detection of PPIs and enzyme-driven PTMs at the physical limit with an organic field-effect transistor platform.

## Introduction

Protein post-translational modifications (PTMs) are one of the most important regulatory mechanisms in cells. PTMs increase the functional variety of proteins and peptides. At present more than 400 PTMs are known to exist in mammalian cells,^1^ but the knowledge of their function is still minimal.^2^ The essential PTMs and their primary functions are phosphorylation (activation/deactivation of enzyme activity), acetylation (protein stability), and methylation (regulation of gene expression).^3^ PTMs play vital roles in cell signaling, and therefore, PTM-catalyzing enzymes have become significant targets for drug discovery. To assay and monitor several different PTMs with a single detection platform would be of great importance in drug development.

Tremendous progress has been made in advancing technologies that identify proteins, measure their concentration in a specimen, and determine their abundance in cells, tissues, and biofluids. This is of potentially very high impact on the early diagnosis of progressive diseases such as cancer. Mutated genes, proteins, and peptides secreted from a diseased cell are termed markers. Hence, marker detection, particularly in peripheral biofluids, and their correlation with a disease’s appearance and growth can be of great relevance for early and minimally invasive diagnosis. Also, peptides have been frequently identified as suitable markers for several progressive diseases. For instance, they have been successfully used to discriminate between cancerous and normal endometrial tissues.^4^ Other applications involve peptides as hormonal markers of ventricular systolic and diastolic dysfunction and ventricular hypertrophy.^5^ Also relevant is the case of the gastrin-releasing peptide specifically secreted by lung carcinoma cells^6^ and the increase in natriuretic peptides after myocardial infarction as a marker of cardiorenal function.^7^

From this perspective, it is paramount to develop ultra-sensitive biochemical methods to monitor peptides, proteins, and their PTMs as markers of progressive diseases. The gold standard in analytical proteomics today, namely mass spectrometry and chromatography, can detect markers, at best, down to 10^−15^ mol · L^−1^ (fM),^8^ translating into hundreds of thousands of markers in a sample volume of 100 μL. This is far from single-molecule detection, which instead requires bioassays capable of discriminating one single binding event from the noise level. In terms of the performance level of this technology, it is therefore critical to settle the bioassay decision threshold, namely the limit of detection (LOD), down to 10–20 zeptomolar (zM; 10^−21^ M), corresponding to 1 ± 1 molecule in 100 μL inspected volume.^9^ The ultimate goal is to detect markers at the single-molecule level, generally achieved using techniques such as recognition/electron tunneling,^10^^,^^11^ nanopores,^12^^,^^13^ and nano-oscillators.^14^ While it is very interesting to study single peptide-peptide interactions, these approaches are not helpful in detecting a single marker in a bulk milieu of 10–100 μL.^15^ Fabrication and scaling of nanotransducers are also very challenging and not reliable enough for commercial applications, particularly high-throughput ones.

Recently, millimeter-sized electrolyte-gated organic field-effect-transistor (EG-OFET) analytical sensors, termed single molecule with a large transistor (SiMoT) technology,^15^^,^^16^ have been proven to exhibit high selectivity and sensitivity, reaching detections in the zeptomolar range. This is a nearly 10^6^-fold lower LOD than mass spectrometry or nanometric interfaces. Interestingly, the biofunctionalization strategy^17^ proposed to functionalize the transducing electrode with biorecognition elements makes the SiMoT platform suitable for detecting different protein and genetic markers at the single-molecule level in real biofluids.^18^^,^^19^

In this work, the SiMoT technology is applied to prove the selective detection of peptides at the physical limit and demonstrate a functional enzymatic PTM detection assay. To this end, the leucine zipper (LZ) interaction is used as a model system for single-peptide detection. LZs are characterized by a common three-dimensional structural motif consisting of a characteristic 30-amino-acid sequence known to act as an enhancer-binding site in proteins.^20^ A self-assembled monolayer (SAM) of the LZ-affinity peptide endows the SiMoT platform with recognition capabilities. The single-peptide binding is hindered when a site-phosphorylated LZ-Y peptide is engaged in the LZ binding.^21^^,^^22^^,^^23^

To the best of our knowledge, no alternative methods are available to measure PTMs using such a label-free, fast, and cost-effective assay. Moreover, given the generality of the approach used in the biofunctionalization strategy of the detecting interface, the SiMoT platform is suitable for detecting a wide spectrum of PTMs. Although mass spectrometry can measure multiple PTMs, this approach is still limited by the low throughput, high cost, and lengthy procedure. The current label-free electronic reading platform represents a first successful proof of concept of a bioanalytical tool capable of detecting down to 10 zM peptide concentration in 100 μL with high selectivity, with a time to result of half an hour. Furthermore, a functional enzymatic peptide PTM was measured with the SiMoT technology and analyzed using multivariate data processing based on principal-component analysis (PCA), enabling a research tool for drug discovery and a fundamental understanding of disease etiology at a sensitivity beyond the existing technologies.

## Results and discussion

### Leucine zipper assay

**Figure 1:**
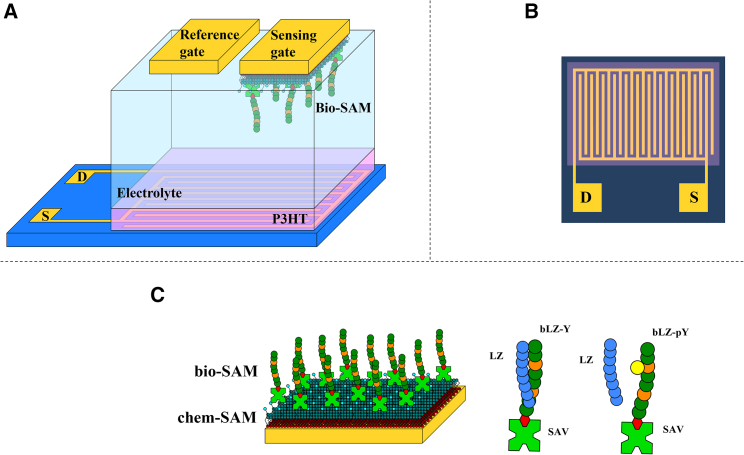
SiMoT platform for LZ assay (A) Three-dimensional schematic view of the SiMoT biosensing device comprising the P3HT electronic channel and the gates (sensing and reference) coupled to the channel via a deionized water electrolyte. They are all immersed in the measuring well. (B) Interdigitated source (S) and drain (D) electrodes characterized by length (L) = 5 μm and width/length (W/L) = 16,000. (C) Schematic representation of the gate surface biofunctionalized with a biotinylated LZ-Y (bLZ-Y) peptide attached to the gate via a streptavidin (SAV) layer. The SAV layer is attached to the gold gate surface via a self-assembled monolayer of mixed alkanethiols (chem-SAM). The peptide binding to be detected (LZ) is also depicted, showing that it binds to bLZ-Y and not to site-specifically modified bLZ-pY.

The SiMoT device, illustrated in Figure 1A, comprises a substrate onto which source (S) and drain (D) gold electrodes have been evaporated. The S-D interdigitated contacts, shown in Figure 1B, are covered by the organic semiconductor poly(3-hexylthiophene-2,5-diyl) (P3HT), forming the electronic channel. The conductivity of the channel is controlled by a gate electrode (G), through deionized water acting as the electrolyte. The device is immersed into a measuring well filled with water (high-performance liquid chromatography [HPLC] grade) serving as a dielectric, where two gates can be hosted. A bare gold gate is used as a reference gate (*vide infra*), while the other is the sensing gate, biofunctionalized with the LZ-complementary peptide. Indeed, to ensure that while the sensing gate is moved from the measuring well to the incubation well the current level in the field-effect transistor (FET) channel is not changed by spurious events, the reference gate is always kept in the measuring well and used to verify the stable operation of the channel. The experimental setting involves peptides specifically designed for protein-protein and protein-peptide LZ interactions in the absence and presence of PTMs. The LZ peptide as a target molecule binds selectively to its LZ-Y affinity peptide pair.^22^ LZ-Y also carries a specific site that enables modifications to be implemented, and site-specific phosphate groups in the tyrosine side chain result in the peptide termed LZ-pY. This peptide is proven to not bind to the affinity peptide immobilized on the sensing-gate electrode, showing that these single-site PTMs can prevent the LZ pairing.^21^^,^^22^^,^^23^ The LZ peptide serves as the analyte or target molecule. To demonstrate a single-peptide detection sensitivity, the peptide LZ-Y is biotinylated (termed hence as bLZ-Y) to enable convenient anchoring to the gate electrode of the SiMoT device. Figure 1C illustrates the fully functionalized gate electrode and the schematic of the LZ binding event. The biofunctionalization protocol foresees the covalent anchoring of the biorecognition elements through an SAM, encompassing short- and long-chain carboxylic-acid-terminating alkanethiols, hereafter termed chem-SAM. The carboxylic moieties of the chem-SAM are then activated through 1-ethyl-3-(3-dimethylaminopropyl)carbodiimide/N-hydroxysulfosuccinimide sodium salt (EDC/NHS) coupling.^15^^,^^24^ This enables conjugating streptavidin (SAV) to the SAM. The biotinylated peptide complementary to LZ, namely bLZ-Y, is immobilized on the sensing gate surface using the biotin/SAV complex formation, holding an extremely high affinity constant (10^15^ M^−1^).^25^ This offers the possibility of stably binding the biotinylated peptide on SAV-modified surfaces.

**Figure 2:**
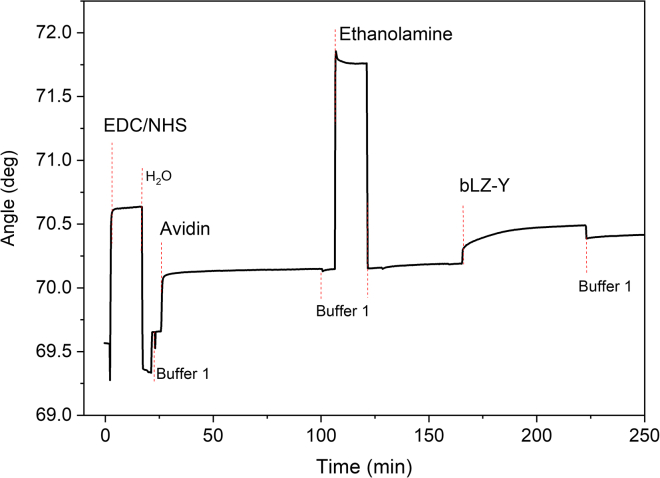
Surface plasmon resonance characterization of the biofunctionalization protocol Sensogram of the immobilization of bLZ-Y on the gold surface premodified with the mixed-alkanethiol chem-SAM and the SAV layer.

The optimization of the biofunctionalization protocol engaged in the SiMoT platform has been used to study the gold surface functionalization *in situ* through a surface plasmon resonance (SPR) Bio-Navi 200-L apparatus equipped with two light sources (670 and 785 nm wavelengths). SPR provides a direct means of tracking molecular-affinity bindings associated with alterations in mass density at the SPR-detecting surface, all without the need for labeling. The active interface, biofunctionalized, is deposited onto a metallic film that coats the optical element’s surface. The optical field of surface plasmons serves as the probe, localized at the active device surface. Variations in the field are quantified by monitoring changes in the local refractive index produced by the formation of biolayers on the sensor surface. In the present study, gold-coated (∼50 nm) SPR slides with a chromium adhesion layer (∼2 nm) were used as the detecting interface. According to the SAM protocol, the gold surface was functionalized with the mixed chem-SAM before SPR measurement. The subsequent functionalization steps were done by static injection of 300 μL of the relevant solutions at 22°C. See Figure 2 for a typical sensogram obtained during all bioconjugation steps of the bio-SAM layer. The amount of biotinylated peptide immobilized per unit area can be estimated from the thickness and the refractive index of a non-homogeneous protein layer based on the Feijter equation, as discussed elsewhere.^26^ The calculated protein surface coverage can be expressed as:

(Equation 1) $$\Gamma =\Delta \theta *550\;ng/{cm}^{2}\text{.}$$

**Figure 3:**
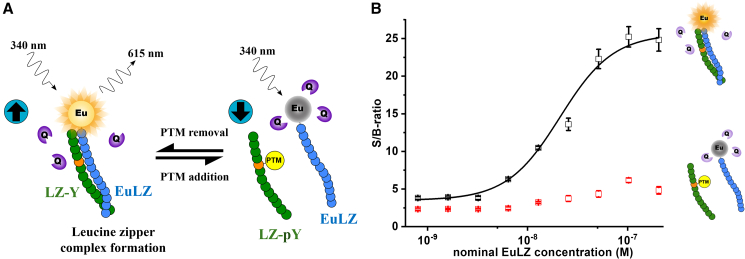
Optical detection of LZ assay (A) Schematic representation of the peptide-break assay using the optical QRET detection. Unmodified peptide sequence (blue) coils with the europium chelate (Eu)-labeled peptide (EuLZ), providing high time-resolved luminescence (TRL) signal at 620 nm (left), while the protein post-translationally modified peptide (PTM; green) binding is disrupted, leading to low TRL signal (right) due to soluble quenchers (Q). (B) Binding curves of LZ-Y (black) and LZ-pY (red) as a function of Eu-labeled peptide EuLZ. EuLZ was titrated from 0.8 to 204.8 nM with 10 nM LZ-Y and LZ-pY using varying concentrations of soluble quenchers to adjust for similar low background signals of the increasing EuLZ concentration, and thereafter the measured TRL signals were normalized to TRL signals without the peptide substrate to obtain the signal-to-background ratios (S/B). The LZ-Y has a distinct response to the increasing EuLZ concentration, whereas LZ-pY remains relatively unresponsive. Data represent mean values ± SD (n = 3).

An angular shift is recorded when 100 μL of a 0.5 μM bLZ-Y buffered solution is injected, as seen in Figure 2. Using Equation 1, the resulting surface coverage Γ = 119 ± 5 ng/cm^2^ is calculated at equilibrium after removing the bLZ-Y excess. Considering the molecular weight of a single bLZ-Y peptide, the estimated average number of active binding sites available is (1.34 ± 0.06) × 10^13^ molecules/cm^2^. This is a very densely packed recognition layer, as is customary for a SiMoT device, as the biorecognition elements are packed at a density of 10^4^ μm^−2^. Moreover, the LZ-Y target peptide exhibits a molecular weight of less than 10 kDa. Consequently, the SPR direct-assay configuration, due to the sensitivity limitations inherent in the technique, proves unsuitable for the detection of small molecules. It worth noting that the calibration curve registered with the SPR technique should have been executed across a substantially elevated concentration range, specifically within the range of 0.1–2 μM, to facilitate accurate measurements.^27^ Consequently, a label-based luminescence binding assay involving an optical readout was carried out to prove that all the peptides perform as expected. Usually, the LOD for luminescence-based assays is between the picomolar and the nanomolar range (10^−12^ to 10^−9^ M).^28^^,^^29^^,^^30^ The peptide interaction study is based on the peptide-break technology harnessed with optical quenching resonance energy transfer (QRET) detection.^21^^,^^22^^,^^23^^,^^31^^,^^32^ In the assay, one of the peptides is labeled with a europium (Eu) (III) chelate, and the luminescence signal of free unbound EuLZ, labeled with a nonadentate (9d) Eu chelate, is quenched in solution with soluble quencher molecules (Q). As shown in Figure 3A, due to the peptide complex formed between the peptides EuLZ and LZ-Y, the luminescence signal is protected from the soluble quenchers, and a time-resolved luminescence (TRL) is observed. When phosphate groups (PTMs) are introduced onto the tyrosine residues of the LZ-Y sequence (LZ-pY), no binding of EuLZ and LZ-pY is observed, due to the interfering phosphate groups that disrupt the binding, leading to luminescence quenching of the unbound EuLZ.

**Figure 4:**
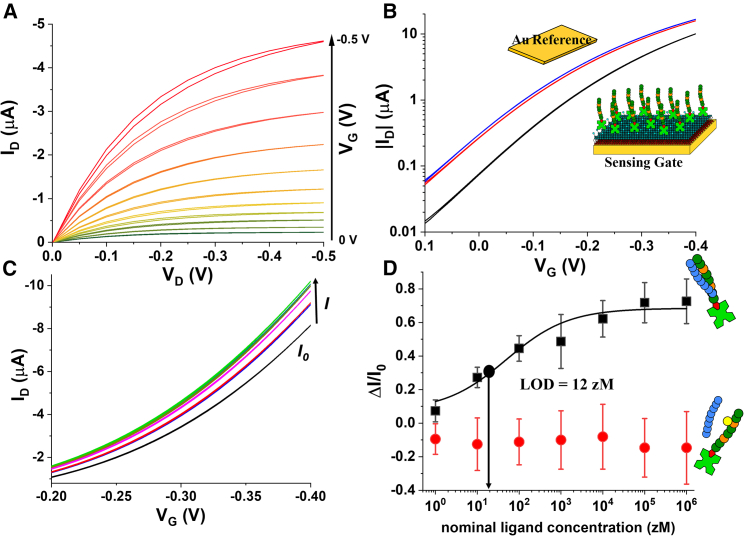
SiMoT electrical measurements (A) Typical output characteristic for a P3HT SiMoT comprising a bLZ-Y-functionalized sensing gate. (B) Transfer I-V curves at $V_{D}=-0.4\;V$ of a SiMoT comprising a bare gold reference gate (blue and red curves) or a bLZ-Y-functionalized sensing gate (black curve). (C) SiMoT transfer I-V curves at $V_{D}=-0.4\;V$ measured upon exposure of the same gate to standard solutions of the target peptide LZ at the following concentrations: 10 (red curve), 100 (blue curve), 10^3^ (magenta curve), 10^4^ (dark yellow curve), 10^5^ (green curve), and 10^6^ zM (dark cyan curve). (D) Dose-response curve for peptide LZ (black squares) measured with the sensing gate functionalized with bLZ-Y. The negative-control dose curves involving the sensing gate functionalized with bLZ-pY are displayed as red circles. The data points represent three replicates of the dose curves, while the error bars were calculated as the standard deviation. The response $\Delta I/I_{0}$ was calculated at $V_{G}=-0.3\;V$.

The optical detection of the binding of the EuLZ peptide to LZ-Y and LZ-pY with the optical QRET detection was performed as follows. The luminescence from the peptide complex is measured as a function of EuLZ-peptide concentration (ranging from 0.8 to 204.8 nM), while LZ-Y concentration and LZ-pY were kept constant (10 nM). The soluble quencher concentration was adjusted for every EuLZ concentration to yield a similar low TRL signal. After that, all TRL signals were normalized with EuLZ signals without the complementary peptide at 620 nm to account for any signal deviation due to non-optimal quencher concentration. The results are presented in Figure 3B. At low concentrations of EuLZ, low TRL signals were observed, as no binding occurs. When the EuLZ concentration was increased, high TRL signals were detected with LZ-Y, corresponding to the LZ pairing of EuLZ and LZ-Y. No significant binding was measured for LZ-pY due to the phosphate modification decreasing the binding affinity. This agrees with our previous results, as the binding affinity was optimized to nanomolar scale to distinguish phosphate-containing tyrosine sequences from the non-modified peptides. The affinity constant of LZ-Y to EuLZ was 21 nM, obtained from the data analysis presented in Figure 3B. The data with the given peptide sequences show that peptides’ subnanomolar concentrations cannot be measured with QRET detection. On the other hand, the SiMoT platform was undertaken to prove subfemtomolar peptide detection. Typical I_D_-V_DS_ output curves for the SiMoT device with a bLZ-Y gate are shown in Figure 4A. The I_D_ has been measured as a function of V_DS_ at different V_G_, varying in the 0 to −0.5 V range. Importantly, the proposed biosensor functions as a potentiometric sensor, meaning that there is no electronic-faradic current involved. Therefore, the operational gating potentials should cover ranges where all the materials in the device remain redox inert. The accumulated charges in the semiconductor generate the output I_D_ current, flowing from the S to the D under a V_DS_ bias. During this phase, when there is no current flow (I_G_) between the gate and the S/D electrodes, I_D_ can be measured.^33^ A good field-effect current modulation has been achieved with negligible hysteresis. The I_D_-V_G_ transfer curves at a fixed V_DS_ = −0.4 V are shown in Figure 4B. Remarkably, each measurement in Figure 4 shows the signal measured after the initial stabilization of the device. Specifically, the blue curve in Figure 4B is the current measured with the reference gate before the sensing measurements. The red curve is the current measured on the same device with the same reference gate after measuring a whole dose-response curve, as shown in Figure 4C. The assessment of the SiMoT current level measured with the reference gate serves to assess that the I_D_ fluctuations fall below 5%. The black curve in Figure 4B shows the I_D_-V_G_ curve when the gate is functionalized with a bLZ-Y SAM. The I_D_ current flowing in the transistor channel in the saturation regime is described by the following equation:^34^

(Equation 2) $$I_{D}=\frac{W\mu_{FET}C_{i}}{2L}{\left(V_{G}-V_{T}\right)}^{2}\;for\;\left|V_{D}\right|>\left|V_{D}^{sat}\right|\text{,}$$

where *W* and *L* are the channel width and length, respectively, while *C*_*i*_ is the gating system capacitance per unit area. *V*_*T*_ is the EG-OFET threshold voltage, while μ_FET_ is the field-effect mobility. According to the above equation, $\sqrt{I_{D}}$ is linear in (*V*_*G*_ − *V*_*T*_), with *V*_*T*_ being the intercept to the *V*_*G*_ abscissa, while (*C*_*i*_*⋅μ*_*FET*_) is proportional to the slope of the linear segment of the curve. The threshold voltage shows a change in the gate electrochemical potential or the work function due to surface functionalization. We observed a *V*_*T*_ of (−62.39 ± 0.13) mV with the bLZ-Y-functionalized gate, while the gold reference gate shows a *V*_*T*_ of (−2.14 ± 0.11) mV. During the sensing measurements, the gate current I_G_ was monitored (not shown) to ensure that the gate current was at least two orders of magnitude lower than I_D_.

Electronic transduction relies on the shift of the gate work function occurring when the binding event between the affinity peptides occurs. This can be measured as an amplified S-D current via capacitive coupling between the sensing gate and the electronic channel in an electrolyte that is an ionic conductor and an electronic insulator. By applying the gate bias, transient ionic currents accumulate ions at the gate/electrolyte and the electrolyte/semiconductor interface responsible for accumulating a channel at the electrolyte/organic semiconductor interface. The charge double layers formed hold capacitances of tens of μF cm^−2^. In Figure 4C, we show the measured transfer curves of the sensing gate electrode, biofunctionalized with bLZ-Y, incubated in buffer solutions containing an increasing concentration of the target peptide LZ. The black curve is the “baseline” (I_0_), which measures the biofunctionalized gate incubated in the buffer solution without the target peptide. No I_D_ current change was measured upon incubation even in the 1 zM target solution. This nominal ligand concentration corresponds to an average of 0 molecules in the sampled solutions. The red curve was recorded upon incubation in a solution of the target peptide at a concentration of 10 zM, corresponding to 1 ± 1 molecule in a 100 μL volume. Indeed, 100 μL of a 10 zM solution can host 0, 1, or 2 analyte molecules (1 ± 1), where the uncertainty associated with the sampling in the serial dilution can be estimated, according to the Poisson distribution, as the square root of the expected number of ligands corresponding to 1 standard deviation.

Remarkably, a relative change in the current of about 25% was registered already at a concentration of 10 zM, as shown in Figure 4C. The response saturation had been achieved already at a nominal ligand concentration of 10^4^ zM (10^−17^ M, 10 aM). In accordance with the customary protocol of the SiMoT assay, the constrained dynamic range observed in the electronic signal implies a binary-type response. It is important to emphasize that the SiMoT assay is intentionally designed for qualitative discrimination rather than quantitative measurement. Its primary purpose is to effectively distinguish between a sample devoid of any peptides and one containing at least one. The qualitative output from the SiMoT platform is inherently linked to the biochemical amplification mechanism, resulting in a significant shift in the whole gate work function. This shift is likely attributed to collective surface phenomena induced by a singular binding event.^40^ As the measured SiMoT sensing signal increases due to LZ binding events, a dose-response curve can be gathered as the anchored bLZ-Y is exposed to increasing concentrations of LZ. The whole dose-response curve for LZ is shown in Figure 4D as black squares. The ΔI/I_0_ dependence from the analyte concentration reported in Figure 4D supports a binary response of the assay, being capable of assessing the total absence or the presence of just one single peptide in the sampled solution. As a control experiment, biotinylated LZ-pY (henceforth bLZ-pY) was attached to the SiMoT gate electrode and exposed to different concentrations of LZ. The phosphate group single-site modification prevents the LZ binding event between LZ and bLZ-pY, which leads to measuring an unchanged SiMoT sensing signal. As shown in Figure 4D (red circles), the negative-control experiment proves that single-site modifications mimicking a post-translational event can prevent label-free detection at the physical limit. No complex formation was measured, as witnessed from the zero electrical response. Each data point plotted in Figure 4D was evaluated as the average of three dose-response curves measured with three different biofunctionalized gates. The relevant error bars were calculated as 1 standard deviation considering that the reproducibility error was within 10%. Moreover, the solid black line is the analytical modeling of the SiMoT sensing response based on Poisson distribution probability^16^ encompassing the following five-parameters logistic equation:

(Equation 3) $$\frac{\Delta I}{I_{0}}=A_{2}+\frac{A_{1}-A_{2}}{1+{\left(\frac{x}{x_{0}}\right)}^{p}}\text{,}$$

where *x* is the LZ concentration, while ΔI/I_0_ is the SiMoT response. The fitting parameters *A*_1_ and *A*_2_ engaged in Equation 3 are, respectively, the initial response for *x* = 0 and the maximum response of the curve, *p* is defined as the Hill coefficient, and *x*_0_ is the inflection point of the curve. The fitting procedure was cross-validated, and the coefficients were adjusted depending on the residual errors in each iteration. A summary of all parameters of the logistic function for the dose-response curve in Figure 4D is reported in Table 1.

**Table 1:** Summary of all parameters of the logistic function (*A*_1_, *A*_2_, *x*_0_, and *p*) for the LZ-Y dose-response curves

Logistic function parameter	Dose response
*A*_1_ = [ΔI/I_0_]^min^ at *x* = 0	0.07 ± 0.01
*A*_2_ = [ΔI/I_0_]^max^	0.68 ± 0.05
*x*_0_	50 ± 28 zM
*P*	0.58 ± 0.16

This accounts for the occurrence of a few binding events, as described in detail elsewhere.^35^ Considering the noise level, namely the average and the standard deviation of the negative-control experiment (red circles data in Figure 4D), an LOD level of $\Delta I/I_{0}=0.29$ has been estimated. From the sensing curve in Figure 4D, the LOD level matches an LZ nominal concentration of 12 zM. Hence, a measurable signal is produced when as low as 1 ± 1 target LZ/bLZ-Y complexes are formed. Therefore, a single LZ is detected to one of the bLZ-Y peptides attached to the gate surface in 100 μL, diffusing and eventually impinging in the minute time scale on a millimeter-wide (e.g., ∼0.6 cm^2^) surface populated with trillions of recognition elements. Importantly, large-area or wide-field transducing interfaces, exposing a much larger active area with respect to near-field biosensing approaches, have been demonstrated to be a viable solution to overcome the diffusion barrier issue.^36^ This is proven by several published experimental results involving mostly FET detections at a large-area interface.^37^^,^^38^^,^^39^ The elicited pieces of evidence gathered on completely different FET structures by several research groups^9^ demonstrate that the diffusion-barrier issue, impairing the use of single-molecule detection at a nanometric interface in assay solution with concentrations below picomolar, does not apply to FET bioelectronic sensors comprising a micrometric or a millimetric wide detecting interface. Moreover, these experiments demonstrate the selectivity of the peptide interaction with the complementary peptide. Hence, combining the SiMoT platform and peptide modification assay enables high selectivity sensing toward single-point modifications at the single-peptide level. Therefore, the SiMoT platform has been proven capable of assaying site-specifically modified and mutated peptide sequences.

It is worth noting that the signal measured when a single LZ peptide is recognized by one of the 10 trillion recognition peptides attached to the gate is higher than the noise level. Such an occurrence is ascribable to the amplification mechanism that is connate with the SiMoT platform. From one side, the amplification originates from the field-effect capacity coupling estimated to be about 10^3^ in SiMoT EG-OFETs.^33^ An additional amplification effect involving an electrostatic domino-like propagation of the electrostatic (dipole) change has been assumed.^15^^,^^33^ Such propagation of a dipolar electrostatic reorientation involving billions of dipoles (capturing antibodies), triggered by one binding event and affecting a sizable area of the millimeter-wide transducing surface, was independently demonstrated using Kelvin probe force microscopy (KPFM).^40^ In particular, the measured work function shift, registered with KPFM upon exposure of the detecting interface to 10 ligands, is correlated to the threshold voltage shift registered with the SiMoT platform. Therefore, those pieces of evidence provide direct experimental confirmation of the SiMoT ultra-high sensitivity by imaging the extensive shift of the gate work function arising from the elicited collective surface phenomena.

### PTM assay

**Figure 5:**
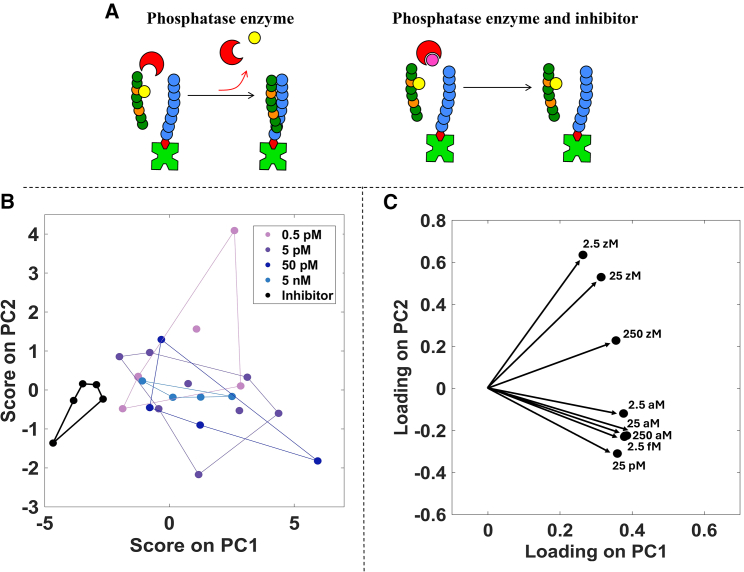
Multivariate data processing of the PTM assay (A) Schematic PTM assay, where the PTP1B enzyme acts on LZ-pY in solution to form detectable LZ-Y, while enzyme inhibition with Na_3_VO_4_ keeps LZ-pY intact. For PTM sensing, bLZ is used as the binding capture peptide functionalized on the sensing gate. (B) The dose-response curve for the score plot on the plane of the first two principal components, PC1 and PC2. (C) Loading scatterplot, showing the loadings of each original variable on PC1 and PC2.

SiMoT technology was further developed toward a functional PTM assay to utilize ultra-sensitive electrical detection of a peptide with single modifications. In this second case, a functional enzymatic assay is demonstrated, immobilizing a biotinylated LZ peptide (bLZ) on the SiMoT gate electrode. The target molecule in this assay is the LZ-Y affinity peptide produced by the dephosphorylation of LZ-pY induced by the enzyme PTP1B phosphatase, as illustrated in Figure 5A (left). According to the enzymatic PTM detection scheme, the phosphatase acts in solution on the LZ-pY peptide, forming the LZ-Y affinity peptide that binds to the bLZ on the gate. This enabled the measurement of the phosphatase dephosphorylation reaction by cleaving tyrosine phosphate groups of the LZ-pY in solution, providing a concept to detect single-substrate dephosphorylations with electrical detection. The bLZ-functionalized gate detected the phospho-cleaved LZ-Y peptide. The affinity binding between bLZ and LZ-Y was measured as customary with the SiMoT platform as an increase in the I_D_ current level. This configuration is more general and potentially detects enzymatically driven peptide and protein PTMs.^22^^,^^32^ In this case, the SiMoT response registered upon exposure to target solutions prepared from stock solutions comprising PTP1B enzyme concentrations of 0.5 pM, 5 pM, 50 pM, and 5 nM and LZ-pY at a concentration of 25 nM. The PTP1B enzyme has an activity of 2.03 μmol/min/mg (batch specific), leading to a calculated substrate conversion of 9.1 × 10^−6^ μmol with an incubation time of 60 min and enzyme concentration of 0.5 pM (7.5 × 10^−8^ mg). With an assay substrate, e.g., LZ-pY concentration of 25 nM (1 × 10^−5^ μmol), the enzyme activity is sufficient to dephosphorylate all the LZ-pY, even at the lowest used enzyme concentration.

As a negative-control experiment, 1 mM Na_3_VO_4_ was added to the PTP1B solution as an inhibitor in the presence of a concentration as high as 5 nM PTP1B enzyme. Indeed, Na_3_VO_4_, shown as a magenta circle in Figure 5A (right), can bind the PTP1B enzyme and inhibit its activity.^23^ Thus, the enzymatic modification of the LZ-pY is prevented, and the LZ-pY remains intact, leading to no significant ΔI/I_0_ change in the SiMoT response.

The sensing protocol engaged in the single-peptide SiMoT assay was then applied to the PTM detection. To this end, starting from the solutions encompassing LZ-pY at a concentration of 25 nM along with PTP1B enzyme concentrations of 0.5 pM, 5 pM, 50 pM, and 5 nM, 10-fold serial dilutions were prepared in the buffer solution. Therefore, nominal ligand concentrations, ranging from 2.5 zM to 25 pM, were inspected, and the SiMoT relative current change ΔI/I_0_ was registered. According to the elicited protocol, the SiMoT relative current change was also evaluated for the negative-control experiment performed in the presence of the inhibitor. All the ΔI/I_0_ values for the whole set of 30 enzymatic reactions are given in Table 2. A minimum of four replicates for each PTM assay with the different concentrations of the PTP1B enzyme were performed.

**Table 2:** List of the PTM assays performed on 30 enzymatic reactions in the presence of Na_3_VO_4_ inhibitor and with PTP1B enzyme, at concentrations of 0.5 pM, 5 pM, 50 pM, and 5 nM

Enzymatic reaction	Enzyme	2.5 zM	25 zM	250 zM	2.5 aM	25 aM	250 aM	2.5 fM	25 pM
1	inhibitor + 5 nM enzyme	−0.07	−0.06	−0.09	−0.08	−0.05	−0.06	−0.06	−0.06
2	inhibitor + 5 nM enzyme	0.01	0.03	0.04	0.05	0.05	0.06	0.06	0.06
3	inhibitor + 5 nM enzyme	0.01	0.01	0.01	0.02	0.02	0.02	−0.09	−0.16
4	inhibitor + 5 nM enzyme	−0.01	−0.01	−0.02	−0.03	−0.04	−0.04	−0.05	−0.08
5	inhibitor + 5 nM enzyme	0.03	0.03	0.02	0.01	0.02	0.02	0.02	0.03
6	inhibitor + 5 nM enzyme	0.07	0.09	0.10	0.06	0.06	0.06	0.09	0.01
7	0.5 pM enzyme	0.13	0.22	0.32	0.30	0.30	0.26	0.26	0.26
8	0.5 pM enzyme	0.02	0.04	0.05	0.18	0.09	0.10	0.19	0.13
9	0.5 pM enzyme	0.01	0.51	0.36	0.25	0.22	0.37	0.33	0.31
10	0.5 pM enzyme	0.05	0.09	0.16	0.16	0.14	0.15	0.13	0.12
11	0.5 pM enzyme	0.06	0.12	0.17	0.22	0.27	0.31	0.34	0.33
12	0.5 pM enzyme	0.02	0.04	0.05	0.18	0.09	0.10	0.19	0.13
13	0.5 pM enzyme	0.05	0.09	0.16	0.16	0.14	0.15	0.13	0.12
14	0.5 pM enzyme	0.06	0.12	0.17	0.22	0.27	0.31	0.34	0.33
15	5 pM enzyme	0.04	0.14	0.43	0.32	0.29	0.18	0.32	0.32
16	5 pM enzyme	−0.01	0.45	0.46	0.46	0.49	0.49	0.49	0.49
17	5 pM enzyme	0.07	0.27	0.40	0.74	0.66	0.74	0.55	0.60
18	5 pM enzyme	0.10	0.11	0.16	0.19	0.12	0.12	0.22	0.14
19	5 pM enzyme	0.09	0.20	0.27	0.37	0.49	0.56	0.60	0.72
20	5 pM enzyme	0.03	0.08	0.17	0.28	0.25	0.26	0.25	0.20
21	5 pM enzyme	0.02	0.06	0.11	0.26	0.41	0.51	0.47	0.85
22	5 pM enzyme	0.04	0.07	0.12	0.15	0.21	0.27	0.25	0.26
23	50 pM enzyme	0.07	0.10	0.15	0.34	0.40	0.48	0.48	0.48
24	50 pM enzyme	0.09	0.22	0.37	0.63	0.79	0.99	1.00	1.00
25	50 pM enzyme	0.11	0.17	0.18	0.18	0.18	0.18	0.18	0.19
26	50 pM enzyme	0.05	0.09	0.15	0.17	0.20	0.22	0.24	0.26
27	5 nM enzyme	0.09	0.27	0.41	0.44	0.54	0.52	0.52	0.52
28	5 nM enzyme	0.17	0.58	0.97	0.45	0.40	0.35	0.38	0.31
29	5 nM enzyme	0.03	0.14	0.16	0.18	0.17	0.18	0.09	0.15
30	5 nM enzyme	0.08	0.20	0.35	0.47	0.45	0.46	0.51	0.52

For each enzymatic reaction, the SiMoT relative current changes registered at different concentrations of PTM-produced target peptide LZ-Y, ranging from 2.5 zM to 25 pM, are reported.

Multivariate data processing based on PCA was undertaken on the whole dataset in Table 2, encompassing 30 samples, to highlight graphical clustering among the different PTMs carried out at different concentrations of the PTB1B enzyme.^41^ Specifically, PCA utilizes orthogonal transformations on a set of interdependent variables to generate a new set of variables known as principal components (PCs), which are linearly uncorrelated. The scores of these PCs represent standardized linear combinations of the original variables, weighted by their loadings. This process allows PCA to reduce the dataset’s dimensions by identifying the linear compositions of the original variables that contribute the most to explained variance. By doing so, PCA creates a more concise and interpretable representation of the complex multivariate relationships present. In the analysis summarized in Table 2, PCA was applied to the SiMoT PTM assay, which includes eight features for each sample after autoscaling. These features correspond to SiMoT relative current changes (ΔI/I_0_) observed at various concentrations of PTM-produced target peptide, ranging from 2.5 zM to 25 pM. The plots of the scores and loadings of the first two PCs, performed on an autoscaled dataset in Table 2, are presented in Figures 5B and 5C. The plot of the scores (Figure 5B) performed on the plane of the first two PCs, explaining 91% of the total variance, clearly shows that the data cluster into two separated groups. PC1 efficiently separates the enzymatic reactions in which Na_3_VO_4_ was added to the PTP1B solution as an inhibitor. Those data cluster at negative values of PC1, while positive values of PC1 characterize all the enzymatic reactions performed in the absence of the inhibitor. When considering the loading plot in Figure 5C, it is apparent that the presence of PTM induced by the enzyme, even at the lowest PTP1B phosphatase concentration of 0.5 pM, results in higher values of PC1. Moreover, the concentration of LZ-Y produced by the enzymatic reaction is explained by PC2. The samples with positive scores on PC2 are characterized by a higher load of the LZ-Y concentrations in the zeptomolar range, while negative scores on PC2 are characterized by a higher load of LZ-Y at concentrations in the attomolar to picomolar range. Moreover, the score plot indicates no significant differences in the conversion efficiency among the different enzyme concentrations, shown in different colors in Figure 5B. Indeed, the electrical detection of the enzymatic reaction utilizes the single-molecule sensitivity of the SiMoT platform. Hence, it is sufficient that a single enzymatic reaction occurs, and the analyte molecule reaches the sensing gate for the detector to respond. Specifically, the sensitivity of the sensor enables it to detect a single peptide that has undergone an enzymatic reaction.

The electrical detection of peptides at the physical level is demonstrated and can be used to detect peptide-peptide or protein-protein interactions, e.g., for diagnostic purposes, once a known affinity pair can be designed. Such a profound sensitivity would greatly benefit drug screening for enzymes controlling PTMs. The SiMoT platform offers the advantage of ultra-sensitive electronic detection, overriding by far the traditional mass-spectrometry (10^6^-fold improvement) or luminescence assays (10^12^-fold improvement). Therefore, the amount of enzyme, typically the most expensive part of the assay, can be reduced. Moreover, SiMoT technology requires a time to result of about 30 min per assay. This encompasses a first incubation step of the sensing gate of about 10 min in the reaction buffer, followed by the baseline acquisition, requiring about 5 min. The sensing gate can therefore be incubated for 10 min in the PTM solution to be assayed, and signal is registered, cycling the SiMoT device with 20 subsequent transfer characteristics for 5 min. Remarkably, the SiMoT approach does not require any sample pretreatment, opening to the way for ultra-high-performing point-of-care devices for large-scale monitoring of protein PTMs.

In conclusion, the ability of the SiMoT technology to detect peptides at the physical limit using an EG-OFET-based device has been demonstrated. Furthermore, the detection of single-site modifications and enzyme-driven PTMs was also demonstrated. The proposed platform successfully demonstrates a bioanalytical tool capable of selectively detecting the given peptides and PTMs at zeptomolar concentration. Alternative protein PTMs can also be measured as a single peptide with single-site sequence modifications. This requires the solution-based peptide modification to match the enzyme specificity while the capture peptide remains unchanged. PTMs detected at the physical limit provide a means to significantly reduce the cost associated with PTM assays, since the enzyme is typically the most expensive component in the assays. As demonstrated in the current study, single peptides can be detected, leading to subpicomolar use of the enzyme in the assay to detect single PTMs, as proven by the multivariate data processing herein undertaken. Therefore, the SiMoT platform could have a significant impact on fundamental research in bioanalytical chemistry, pushing label-free bioassay detection performance toward the physical limit without losing selectivity. The method is highly valuable for early and minimally invasive diagnosis and drug-discovery applications.

## Experimental procedures

### Resource availability

#### Lead contact

Further information and requests for resources should be directed to and will be fulfilled by the lead contact, Ronald Österbacka (ronald.osterbacka@abo.fi), upon reasonable request.

#### Materials availability

This study did not generate new unique materials.

#### Data and code availability

The data generated in this study are included in the article and will be made available from the lead contact upon reasonable request.

### Chemical reagents and materials

The peptides used in this study were acquired from Pepmic (Suzhou, China), and their amino acid sequences and molecular weights are summarized in Table 3. The peptides were commercially acquired from a reliable source with known mass and molecular weight to obtain concentrations. Peptides were directly dissolved in the original tube to avoid any error related to the weighing of small quantities. Recombinant human PTP1B and the inhibitor Na_3_VO_4_ were purchased from Thermo Fisher Scientific and MP Biomedicals, respectively. All the other chemicals were purchased from Sigma-Aldrich (St. Louis, MO, USA). P3HT(regioregularity >99%, M_w_ 17.5 kDa), 3-mercaptopropionic acid (3-MPA), 11-mercaptoundecanoic acid (11-MUA), EDC, NHS, SAV from *Streptomyces avidinii* lyophilized from 10 mM potassium phosphate, HPLC-grade water, and ethanol grade puriss. p.a. assay, ≥99.8%, were used with no further purifications unless otherwise stated. Phosphate-buffered saline (PBS) solution, holding physiological osmolality and ion concentration, was prepared according to the standard protocol reported elsewhere.^15^ PBS thus serves to reproduce a physiologically relevant fluid with a pH of 7.4 and ionic strength of 162 mM, mimicking the environment of blood serum. The reaction buffer for peptide interaction studies was 10 mM HEPES, 1 mM MgCl_2_, 0.1 mM EDTA, 5 mM NaCl, and 0.01% Triton (pH 7.4), at 25°C.

**Table 3:** Peptide sequences

Peptide	Amino acid sequence	Molecular weight (Da)
LZ	KDTLQAETDQLEDEKSALQTEIANLLKEKEKLEFIL	4,176
bLZ	KDTLQAETDQLEDEKSALQTEIANLLKEKEKLEFILGSGSK(biotin)-NH_2_	4,817
LZ-Y	REELRKRRAELRRRYAQLRQRREQLRQRYANLRKE	4,682
bLZ-Y	REELRKRRAELRRRYAQLRQRREQLRQRYANLRKEGSGSK(biotin)-NH_2_	5,324
LZ-pY	REELRKRRAELRRR(pTyr)AQLRQRREQLRQR(pTyr)ANLRKE	4,842
bLZ-pY	REELRKRRAELRRR(pTyr)AQLRQRREQLRQR(pTyr)ANLRKEGSGSK(biotin)-NH_2_	5,484

### SiMoT sensing measurements

Before the sensing measurements were performed, each SiMoT device was stabilized by registering subsequent transfer characteristics (S-D current I_D_ vs. the gate bias V_G_ at a fixed S-D bias V_D_) with a bare gold gate, namely the reference gate, until a stable value was reached. After the initial stabilization, the functionalized gate electrode was incubated for 10 min in 100 μL of the peptide reaction buffer at room temperature in the dark. After incubation, the gate was thoroughly washed with HPLC water and inserted into the measuring well over the channel area, and a set of 20 subsequent transfer characteristics was registered. The stable current I_D_ registered at the V_G_ value that maximizes the transconductance δI_D_/δV_G_ over the whole dynamic range (typically at −0.3 V) was taken as the baseline (I_0_). The same gate was subsequently incubated in 100 μL of the buffer standard solutions of the analyte, with nominal concentrations ranging from 1 to 10^8^ zM (10^−21^ M). The target peptide standard solutions were prepared by a serial dilution according to the following equation: c_2_ = c_1_ · V_1_/V_2_ = k · c_1_. Here c_1_ and c_2_ are the ligand concentrations in the stock and in the diluted solution, respectively, while V_1_ and V_2_ are the corresponding solution volumes, and k = V_1_/V_2_ is the dilution factor. As customary, the former dilution is the stock solution for the subsequent dilution in the series. The absolute uncertainty of the concentration for each standard solution was computed as the propagation error of the dilution factor, while the uncertainty of the volume, given by the supplier company of the pipettes used, was 1%. This value of the volume uncertainty considers both random and systematic errors in pipetting. Each incubation took 10 min and was performed in a dedicated incubation well. After incubation in each buffer standard solution of the target peptide, the gate was rinsed with water to remove unbound species before being replaced in the measuring well to record the transfer curves. Starting from more diluted standard solutions, a further set of transfer characteristics was recorded, and the stable I_D_ at maximum transconductance was taken as the signal I at that particular concentration. A non-regenerative approach was undertaken due to the irreversible nature of the biorecognition element/ligand binding. Indeed, gold gate surface regeneration between consecutive analyte injections to remove the bound peptides cannot be foreseen in the case of stable ligand-analyte complexes, such as peptide-peptide interactions involved in this study. The normalized current change at a given concentration obtained as ΔII0=I−I0I0, was taken as the SiMoT response. As a negative-control experiment, the SiMoT sensing gate was biofunctionalized with the non-binding biotinylated peptide bLZ-pY, exposed to LZ peptide following the protocol previously described.

The monitoring of the enzymatic reaction was accomplished with a SiMoT sensing gate biofunctionalized with biotinylated bLZ, incubated with standard solutions encompassing 0.5 pM to 5 nM concentrations of the PTP1B enzyme that dephosphorylates LZ-pY. Therefore, the LZ-Y analyte produced by the enzyme is then capable of binding to the SiMoT sensing gate. The negative-control experiments were performed in the presence of Na_3_VO_4_ to inhibit the enzymatic reaction, thus preventing dephosphorylation and subsequent binding to the sensing gate. All the data were averaged over at least three replicates, measured on three different SiMoT devices. The resulting reproducibility error was computed as the relative standard deviation. The LOD was computed as the concentration that corresponds to an average response of the blank sample plus three times its standard deviation.

### SiMoT device fabrication

The SiMoT devices were fabricated starting from silicon substrates with gold S and D interdigitated electrodes, purchased from the VTT Technical Research Centre of Finland, with channel length L = 5 μm and channel width W = 80,000 μm, thus resulting in a W/L ratio of 16,000. The electrode-covered substrate was sonicated in 2-propanol before depositing the organic semiconductor P3HT.^42^ The P3HT concentration in solution was 4 mg/mL, and it was spin-coated at 2,000 rpm for 30 s and further annealed at 90°C for 60 min in darkness. Then the channel area was covered with a polydimethylsiloxane well filled with 300 μL of HPLC-grade water, being the maximum volume that could be hosted by the well glued on top of the electronic channel. This served as the electrolyte solution. A Kapton foil covered with an electron-beam evaporated gold (50 nm) on titanium (5 nm) with an area of ∼0.6 cm^2^ was used as the gate electrode. The gate area was chosen to be significantly larger than the interdigitated S-D area, and the volume of electrolyte was chosen to be large enough to host both the biofunctionalized gate and the reference gate electrodes. The gate was designed to be larger than the P3HT electronic channel, so that the low capacitive coupling between the gate and the organic semiconductor turned into an FET that is modulated very well by the charge variations occurring on the gate electrode. In other words, the device is by design extremely sensitive to electrostatic changes in the biolayer, thus triggering a threshold voltage shift upon interaction of the biolayer with the cognate peptide. The gate was positioned in the water on top of the interdigitated electrode area at about 3 mm from the channel area. P3HT is compatible with cost-effective large-area printing techniques, such as ink-jet printing, and possibly fabricating printed devices on flexible and sustainable substrates. All the device-to-device fluctuations between the different sensing experiments conducted on different gates and different EG-OFETs normalized as the relative current changes, used as the SiMoT response, were evaluated as the change in the D current normalized for each experiment.

### SiMoT gate biofunctionalization

The gate electrodes were sonicated in 2-propanol for 10 min and subsequently rinsed with HPLC water, dried with N_2_, and then treated for 2 min using an ozone cleaner. The gate biofunctionalization protocol is described in detail elsewhere.^17^ First, a chem-SAM is immobilized on the gold surface. The chem-SAM consists of mixed carboxylic terminated alkanethiols that generate a dense coverage on a gold surface. The bio-SAM is subsequently attached to the chem-SAM, using EDC and NHS chemistry. Subsequently, the activated gate surface is incubated in a PBS solution of SAV (0.1 mg mL^−1^), while the unreacted NHS groups are deactivated with ethanolamine. As the final functionalization step, the gate is immersed in the biotinylated peptide biorecognition element, completing the bio-SAM through the extraordinarily high binding affinity of SAV to biotin.^25^ The strong biotin-SAV linkage ensures the stability of the LZ-Y biofunctionalization and widens the SiMoT platform deployment to detect different peptides. Moreover, considering the gate electrode area and the molecular weight of the biotinylated peptides, ∼10^13^ capturing peptides are immobilized on the gate surface, as demonstrated through SPR characterization (*vide supra*).

### Ligand standard solution preparation

The peptide standard solutions, at nominal concentrations ranging from 1 to 10^6^ zM (10^−15^ M, 1 fM), were prepared by a serial dilution process with the dilution factor given by c_n_·V_n_ = c_n − 1_·V_n − 1_, where c_n − 1_ and c_n_ are the ligand concentrations in stock and in the diluted solution, respectively, while V_n − 1_ and V_n_ are the volumes of the stock and of the diluted solution. As customary, in a serial dilution process, the former dilution is the stock solution for subsequent dilution in the series. The absolute uncertainty on the concentration for each standard solution was computed as the propagation error of the dilution factor, where the uncertainty of the volume, given by the supplier company of the pipettes used, is 1%. The nominal number of the peptides is computed as c·V·N_A_, where c is the ligand concentration, V is the volume of the standard PBS solution in which the gate is incubated (100 μL), and N_A_ is Avogadro’s number. The uncertainty associated with the sampling in the serial dilution can be estimated, according to the Poisson distribution, as the square root of the expected number of peptides corresponding to 1 standard deviation. The total uncertainty on the ligand concentration was evaluated as the square root of the sum of the squares (RSS) of the dilution (σ_D_) and Poisson’s (σ_P_) errors (both expressed in molarity), hence, $\sigma_{RSS}=\sqrt{\sigma_{D}^{2}+\sigma_{P}^{2}}$. The relevant error bars, used throughout the present study, are shown in Table 4.

**Table 4:** Ligand solutions along with the relevant error bars used in this study

[Peptide] (zM)	σ_RSS_ (zM)	No. of peptides	σ_P_ (n)
1	0^a^	0	0
10^1^	5	1	1
10^2^	33	6	2
10^3^	0.4 × 10^2^	62	8
10^4^	0.1 × 10^3^	623	24
10^5^	0.04 × 10^4^	6.23 × 10^3^	79
10^6^	0.02 × 10^5^	6.23 × 10^4^	2 × 10^2^

a The error is taken as the sole Poisson one.
